# A Malaysian Delphi consensus on managing knee osteoarthritis

**DOI:** 10.1186/s12891-021-04381-8

**Published:** 2021-06-04

**Authors:** Swan Sim Yeap, Syamsul Rizal Abu Amin, Hazlyna Baharuddin, Kar Chai Koh, Joon Kiong Lee, Verna Kar Mun Lee, Nor Hamdan Mohamad Yahaya, Cheh Chin Tai, Maw Pin Tan

**Affiliations:** 1 Department of Medicine, Subang Jaya Medical Centre, No. 1, Jalan SS12/1A, 47500 Subang Jaya, Selangor Malaysia; 2 Sport Medicine Unit, Hospital Selayang, 68100 Batu Caves, Selangor Malaysia; 3 Department of Internal Medicine, Universiti Teknologi MARA, Kampus Sungai Buloh, Jalan Hospital, 47000 Sungai Buloh, Selangor Malaysia; 4 Poliklinik Kepong Baru, Jalan Ambong Kiri Satu, Kepong Baru, 52100 Kuala Lumpur, Wilayah Persekutuan Kuala Lumpur Malaysia; 5 Department of Orthopaedic Surgery, Beacon Hospital, Jalan Templer, Section 51, 46050 Petaling Jaya, Selangor Malaysia; 6 Department of Family Medicine, School of Medicine, International Medical University, No. 126, Jalan Jalil Perkasa 19, Bukit Jalil, 57000 Kuala Lumpur, Wilayah Persekutuan Kuala Lumpur Malaysia; 7 Department of Orthopaedics and Traumatology, Faculty of Medicine, Universiti Kebangsaan Malaysia, Jalan Yaacob Latif, Bandar Tun Razak, 56000 Kuala Lumpur, Wilayah Persekutuan Kuala Lumpur Malaysia; 8 Department of Orthopaedic Surgery, Ara Damansara Medical Centre, Jalan Lapangan Terbang Subang, Seksyen U2, 40150 Shah Alam, Selangor Malaysia; 9 Department of Medicine, Faculty of Medicine, University of Malaya, 50603 Kuala Lumpur, Malaysia; 10 Department of Medical Sciences, Faculty of Healthcare and Medical Sciences, Sunway University, 47500 Bandar Sunway, Selangor Malaysia

**Keywords:** Knee osteoarthritis, Multimodal intervention, Malaysian consensus, Algorithm

## Abstract

**Background:**

The 2013 Malaysian Clinical Practice Guidelines on the Management of Osteoarthritis (OA) recommend a linear step-up approach to manage knee OA. However, patients with knee OA often require a multimodal approach to address OA-related pain symptoms and functional limitations. This consensus aimed to provide doctors with an updated set of evidence-based, clinical experience-guided recommendations to manage knee OA.

**Methods:**

A multi-speciality expert panel consisting of nine Malaysian physicians from different healthcare settings who manage a diverse OA patient population was convened. Using a combination of the ADAPTE process and modified Delphi method, the panel reviewed current evidence on the management of knee OA and synthesised a set of nine recommendations on the management of knee OA, supported by an algorithm that summarises the consensus’ core messages.

**Results:**

A multimodal intervention strategy is the mainstay of OA management and the choice of any single or multimodal intervention may vary over the course of the disease. Overall, a non-pharmacological core treatment set of patient education, weight loss and exercise is recommended for all patients. When pharmacotherapy is indicated, symptomatic slow-acting drugs for osteoarthritis are recommended at the early stage of disease, and they can be paired with physical therapy as background treatment. Concurrent advanced pharmacotherapy that includes non-steroidal anti-inflammatory drugs, intraarticular injections and short-term weak opioids can be considered if patients do not respond sufficiently to background treatment. Patients with severe symptomatic knee OA should be considered for knee replacement surgery. Management should begin with specific treatments with the least systemic exposure or toxicity, and the choice of treatment should be determined as a shared decision between patients and their team of healthcare providers.

**Conclusions:**

This consensus presents nine recommendations that advocate an algorithmic approach in the management of patients living with knee OA. They are applicable to patients receiving treatment from primary to tertiary care providers in Malaysia as well as other countries.

## Background

Osteoarthritis (OA), being the most prevalent form of arthritis, is characterised by progressive cartilage loss with remodelling of adjacent bone, leading to structural changes of the joint, causing pain and functional disability [1]. The pathophysiology of OA includes a slow but inefficient repair process that often compensates for the initial trauma/damage, resulting in a structurally altered but symptom-free joint. When this process fails to compensate (“joint failure”), because of either overwhelming trauma or compromised repair, patients will present with symptomatic OA [2].

Based on the Global Burden of Disease 2010 study, OA of the hip and knee was the 11th highest contributor to global disability, and the global age-standardised prevalence of knee OA was 3.8% (95% uncertainty interval, 3.6 to 4.1%) [1]. Severe knee pain is an independent predictor of fall risk among the older population [3], and patients with OA have been shown to be at a higher risk of death compared with the general population [4]. Musculoskeletal disorders accounted for 6.3% of Malaysia’s disability-adjusted life years per 100,000 population in 2017 [5]. According to the Community Oriented Programme for the Control of Rheumatic Diseases (COPCORD) study conducted in Malaysia, 64.8% of all joint-related complaints were regarding the knee; among patients assessed for knee pain, over half showed clinical evidence of OA [6].

Consistent with global trends, Malaysia is also facing an increasing prevalence of OA owing to a combination of risk factors. Age and obesity are two major factors that elevate the risk of OA [7], both of which are increasingly prevalent in Malaysia. In 2019, the percentage of the Malaysian population aged ≥65 years was estimated at 6.7% out of 32.6 million people; by 2040, the percentage of the older population is expected to reach 14.5% [8, 9]. In a cross-sectional survey from the Malaysian Elders Longitudinal Research (MELoR) study, knee pain was present in 33.2% of 1212 study participants [3]. According to the 2019 Malaysian National Health and Morbidity Survey, the prevalence of overweight and obesity in the country were 30.4 and 19.7%, respectively [10].

The longitudinal Framingham Heart Study first showed that occupations that combine knee bending and physical demands may be an important contributor of radiographic OA [11]. This observation was confirmed by a cross-sectional study in Japan that associated kneeling and squatting with lower minimum joint space width and higher osteophyte area – structural changes that are commonly seen in those with knee OA [12]. As an Asian country, the practice of kneeling and squatting is also commonly seen among the Malaysian population [13]. A Malaysian study found that squatting exacerbated discomfort and knee pain in patients with knee OA, which disrupted activities of daily living [14].

Collectively, these risk factors contribute to a significant burden of musculoskeletal disorders in the country; musculoskeletal pain (including knee pain) has been reported as one of the most common reasons for patients to seek medical care in the primary care setting [6, 15, 16]. The Malaysian Clinical Practice Guidelines (CPG) on the Management of Osteoarthritis (2nd edition) was published in 2013, but has not been updated since to reflect the latest available evidence on the management of knee OA [7]. The current CPG recommends a linear step-up treatment approach, where each intervention is only introduced upon the failure of the previous therapy, indicated by persistent symptoms that affect patients’ quality of life (QOL).

In the real-world, the growing burden of chronic diseases (e.g., knee OA) highlighted the need for a more ‘proactive’ approach in patient management [17]. Based on the individual needs of each patient, physicians can employ a multimodal approach that utilises a combination of pharmacological and non-pharmacological interventions according to the severity of knee OA. Such an approach prevents the deterioration of patient’s condition and preserves their QOL.

While current international OA guidelines serve as important references that provide evidence-based disease management recommendations, they often lack localised clinical input that optimises the treatment pathway for specific patient populations [18, 19]. Specifically, international guidelines often do not consider the limitations of drug availability and access in individual countries. To address this gap, an expert panel of specialist physicians and surgeons authored this consensus to provide doctors with a set of evidence-based recommendations with an accompanying algorithm, taking into account drug availability, prescribing preferences, and local practices, that can be used to optimise the management of patients with knee OA in Malaysia.

## Methods

An expert panel consisting of nine Malaysian physicians from different specialties with experience in the management of knee OA was convened. The panel was gathered by two senior consultants, namely SSY and JKL, who recruited seven other panellists based on the following criteria: (i) at least 15 years of clinical experience in managing patients with knee OA in their respective practice, (ii) still actively treating patients with knee OA (average 60 visits/month), and (iii) representing the unique different healthcare settings in Malaysia (i.e., primary and hospital care; public, private and academic centres). They included three orthopaedic surgeons (two from private and one from academic centres), two rheumatologists (one each from private and public/academic centres), an academic family medicine specialist, an academic geriatrician, a private general practitioner, and a public sports medicine physician. The panel’s scope of work included developing a set of consensus recommendations and a one-page algorithm on the optimal management practices of knee OA that are relevant to the Malaysian population.

The panel planned to develop a set of evidence-based and clinical-guided consensus using a combination of the ADAPTE process and modified Delphi method [20–22]. SSY and JKL first conducted a literature search on Medline (via PubMed) using the following terms: ‘knee’, ‘osteoarthritis’, ‘management’, ‘guideline’, and ‘recommendation’ to identify knee OA guidelines that could be adapted to the Malaysian setting. From a total of 102 search results, the panel identified and appraised 11 clinical guidelines for their relevance and applicability.

Subsequently, four meetings were held between October 2019 and September 2020. At the first meeting, the panel decided that the 2019 European Society for Clinical and Economic Aspects of Osteoporosis, Osteoarthritis and Musculoskeletal Diseases’ (ESCEO) algorithm recommendation for the management of knee OA would provide the most appropriate foundation for the development of the Malaysian consensus.

During the second meeting, a total of 15 recommendation statements from the ESCEO 2019 guideline were reviewed by the panel. Each original statement was assessed and debated for their relevance to clinical practice in Malaysia, and six recommendations were unanimously rejected by the panel. Using the remaining nine recommendations as anchor points, the panel performed a second literature review using PubMed (https://pubmed.ncbi.nlm.nih.gov/) and Google Scholar (https://scholar.google.com/) to collate evidence that supported each recommendation. Relevant studies published between 2001 and 2020 were identified using these search terms: ‘knee osteoarthritis’, ‘degenerative arthritis’, ‘knee pain’, ‘function’, ‘WOMAC’, ‘treatment’, ‘SYSADOA’, ‘NSAIDs’, ‘paracetamol’, ‘intra-articular injection’, ‘surgery’, ‘consensus’, ‘algorithm’, and ‘Malaysia’.

The search results were then screened using the following criteria: (a) evidence-based management of OA, including pharmacological and non-pharmacological treatment options; (b) complete outcome data as assessed by the Western Ontario and McMaster Universities Arthritis Index (WOMAC), visual analog scale (VAS), Lequesne index, or joint space width (JSW), with comparisons at baseline and end of follow-up periods for all study groups; (c) availability of systematic review, meta-analysis, or randomised controlled trial; and (d) studies in a Malaysian population. Additional studies that were relevant, but not retrieved from the search strategy, were added from the personal files of the panel.

Guided by the compiled evidence (101 published references) and the panel’s own clinical experience, nine Malaysian-centric treatment recommendations were synthesised and an associated algorithm was proposed to supplement the recommendations. Next, individual panellists indicated their respective position on each proposed recommendation using a five-point Likert scale (1 = strongly disagree, 2 = disagree, 3 = neutral, 4 = agree, 5 = strongly agree) by participating in a blinded online voting exercise. Consensus was defined a priori as a combined ‘strongly agree’ and ‘agree’ acceptance rate of ≥70%, with no more than one outlier, or ≥ 1 Likert point from the mean in either direction [23]. Five recommendations (i.e., Recommendations 1–3, 7 and 8) reached a consensus after the first round of voting and were accepted unchanged. The disagreements regarding the other four recommendations (i.e., Recommendations 4–6 and 9) were resolved at the third meeting through discussion and a second round of open voting.

**Fig. 1:**
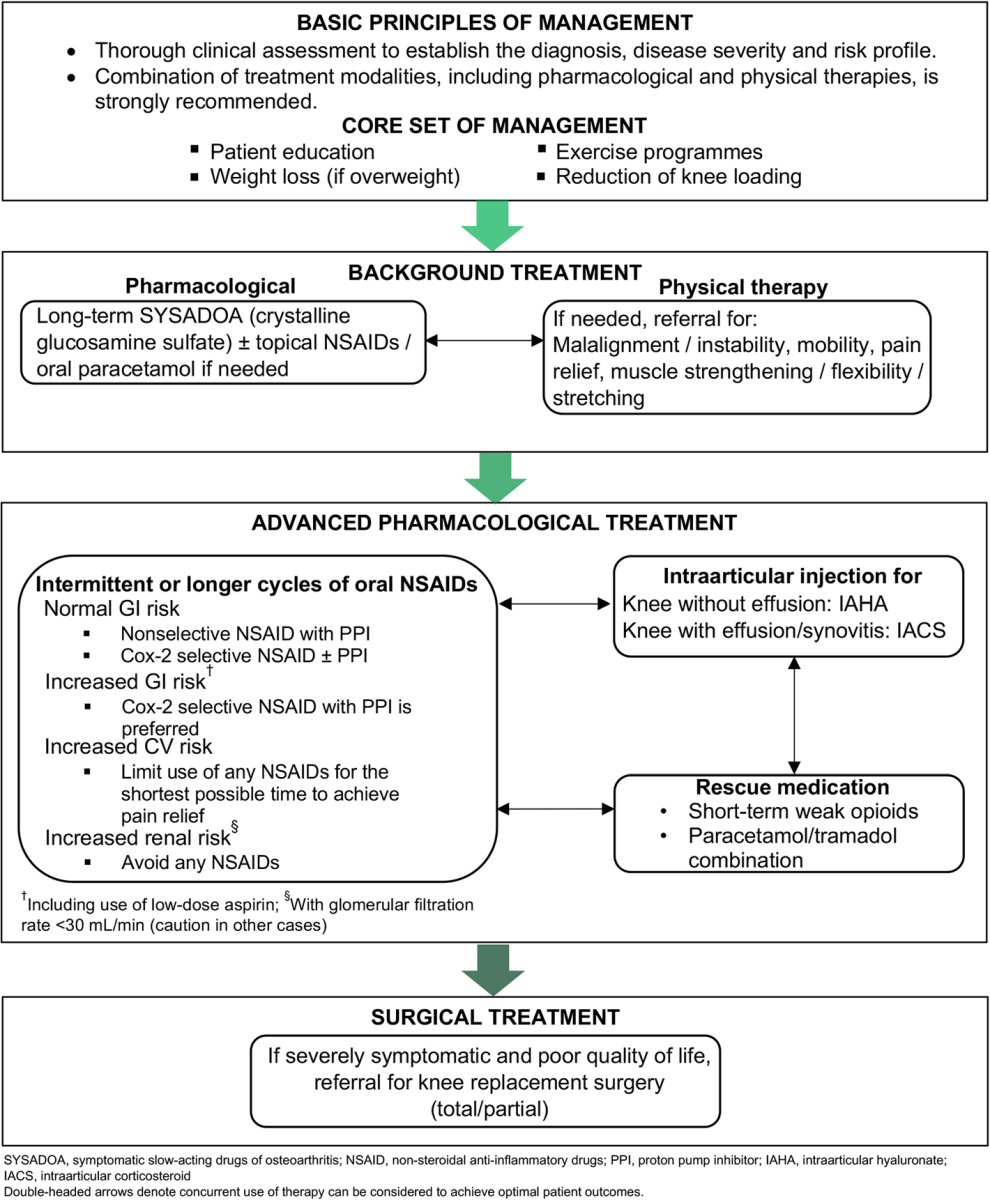
Malaysian consensus on the management of knee OA (2021): Recommended algorithm

At the fourth and final meeting, the panel scrutinised each recommendation, along with their respective elaborations, for scientific accuracy and local relevance. All nine recommendations achieved 100% level of agreement, and the accompanying Malaysian knee OA algorithm was adjusted, until it was unanimously approved, to reflect the disease management principles expounded by the recommendations (Fig. 1).

## Results

### Recommendations of knee OA management

#### Recommendation 1

The group recommends that all patients with osteoarthritis of the knee should be assessed carefully to establish their diagnosis, severity and clinical risk profile before initiating treatment.

Patients who demonstrate symptoms suggestive of knee OA should be assessed carefully at the initial consultation [24]. A comprehensive first assessment is necessary for the implementation of an individualised management strategy to achieve optimal patient outcomes [25]. The initial patient assessment should adopt a biopsychosocial approach and include a review of the individual’s physical status, activities of daily living, health education needs, health beliefs, as well as motivation to self-manage and overall psychological state. Additionally, it is also important to ascertain the presence of cardiovascular (CV) disease and other comorbidities during the initial assessment. These assessments should ideally be repeated at regular intervals during the patient’s follow-up sessions.

**Fig. 2:**
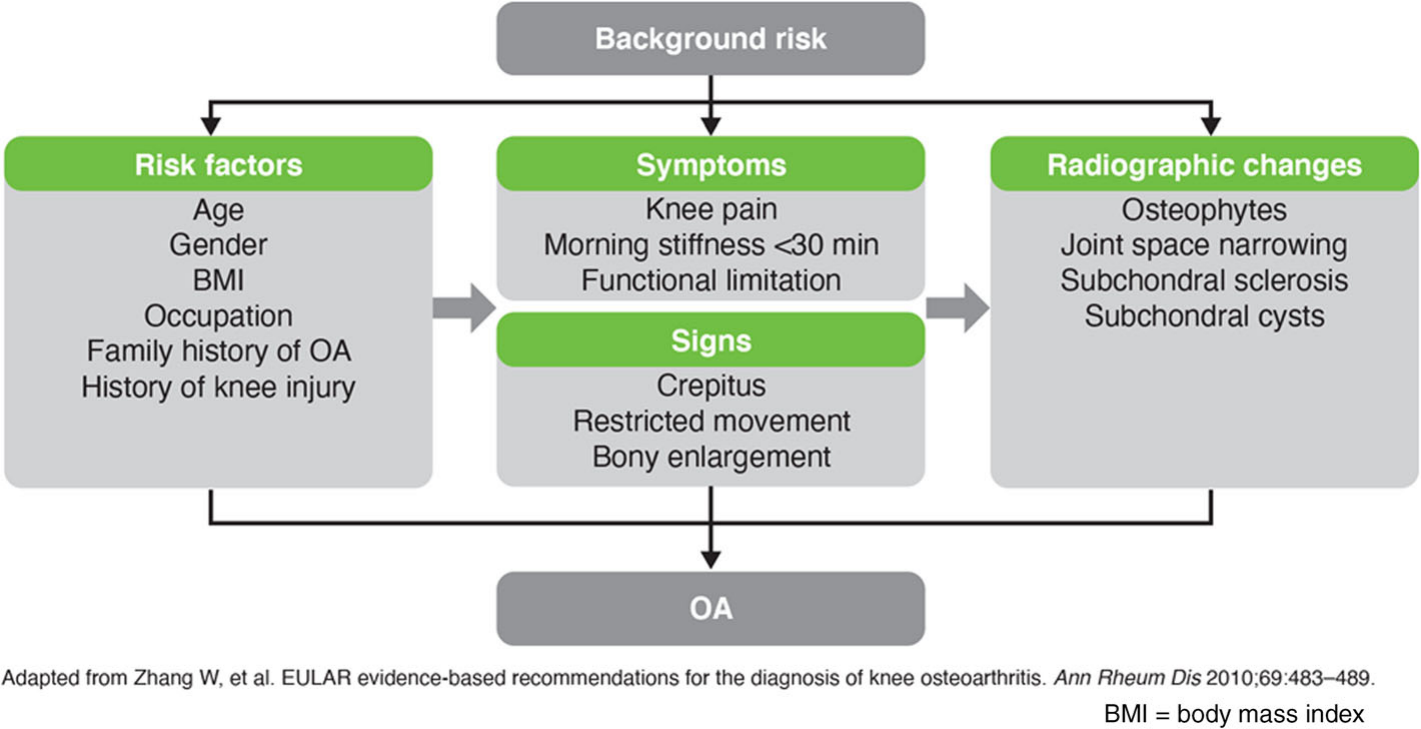
Major components in the diagnosis of knee OA [24]

The diagnosis of knee OA can be made clinically based on patient history and physical examination. Persistent knee pain, morning stiffness of < 30 min, reduced function (especially an inability to squat), together with risk factors strongly associated with the incidence of knee OA such as age > 50 years, female gender, higher body mass index, previous knee injury or malalignment, joint laxity, selected occupational or recreational usage, and a positive family history would be suggestive of knee OA. On examination, the signs suggestive of knee OA include crepitus, restricted range of movement, and bony enlargement (Fig. 2) [24]. Additionally, the presence of deformity, instability, periarticular or joint-line tenderness around the knee, pain on patellofemoral compression, and/or Heberden’s nodes may also be present in those with knee OA.

In terms of investigation, weight bearing x-rays of the knee is accepted as the current ‘gold standard’ for the morphological assessment of knee OA, with focal joint space narrowing (JSN), the presence of osteophyte(s), subchondral bone sclerosis, and subchondral ‘cysts’ being considered classical features of knee OA. However, European League Against Rheumatism (EULAR) recommendations highlighted that plain radiographs should be used as an adjunct as opposed to being a central feature of diagnosis [24]. In adults aged ≥45 years presenting with the above signs and symptoms, a firm diagnosis of knee OA can be made without radiographic assessment or even when the subject’s radiograph appears normal. While blood, urine or synovial fluid tests are unnecessary, they may be used to confirm or exclude coexisting inflammatory disease in patients with suggestive symptoms or signs.

#### Recommendation 2

The group recommends that treatment should be individualised to each patient’s symptoms, and a combination of treatment modalities, including pharmacological and physical therapies, should be used to manage patients with knee osteoarthritis.

According to EULAR recommendations, the individualisation of treatment should be used as the main guiding principle in managing patients with knee OA [24], a position supported by the panel. Specifically, the treatment of knee OA should be personalised as a package of care based on shared decision-making, taking into account the presence of OA risk factors (e.g., age, sex, comorbidity, obesity and adverse mechanical factors), presence of inflammation, severity of structural change, level of pain and restriction of daily activities, along with societal participation and quality of life (QOL).

Randomised controlled trials (RCT) have shown that individualised non-pharmacological management of knee OA resulted in reduced pain (mean difference, 95% confidence interval [CI] [0–20 point scale]: − 1.19, − 2.1 to − 0.3 and − 1.01, − 1.84 to − 0.19) and improved physical function (mean difference, 95% CI [0–68 point scale]: 3.65, 1.0 to 6.3 and 3.33, 0.78 to 5.88) compared with usual care [26, 27].

A comprehensive plan for the management of OA in an individual patient may include educational, behavioural, psychosocial, and physical interventions, as well as topical, oral, and intraarticular medications [19]. In the 2019 American College of Rheumatology/Arthritis Foundation Guideline for the Management of OA of the Hand, Hip, and Knee, it is suggested that various pharmacological and non-pharmacological options may be used (and reused) at various intervals during the course of a patient’s disease [19]. It is important to acknowledge that while some patients may achieve adequate symptom control from a single mode of intervention, others may require sequential or combined use of various interventions to achieve optimal outcomes.

#### Recommendation 3

The group recommends the application of a core treatment set, consisting of patient education, weight loss (if overweight), exercise programmes, and reduction of knee loading throughout the management of knee osteoarthritis.

Core treatments are interventions that are considered suitable for use by most patients in almost any scenario, and are considered safe to use in combination with first and second-line pharmacological treatments. Core treatments for knee OA should include arthritis education and structured land-based exercise programmes in combination with weight management (if needed) [28]. A narrative review that aimed to identify all possible risk factors that can be modified postulated that a holistic approach to the management of knee OA may consist of quadriceps muscle-strengthening exercises, supplemented with a proper diet, weight loss, vocational rehabilitation, management of comorbidities, and orthotics [29].

Patient education is a life-long process that plays an integral role in increasing adherence to exercise and weight loss programmes, thus promoting long-term benefits. Patient education should include appropriate educational information, self-management training, and a suitable medium to convey the information. In Malaysia, patient education is particularly important as the use of traditional and complementary medicine (TCM) for knee pain remains widespread owing to the perceived risks of allopathic medicine. In a cross-sectional survey, 57.9% of patients reported prior use of TCM for knee OA; the use of TCM was higher among males (OR, 2.47; 95% CI, 1.28 to 4.77), and those with a longer diagnosis of OA (OR, 1.51; 95% CI, 1.03 to 2.23) or greater severity of knee pain (OR, 2.56; 95% CI, 1.71 to 3.86) [30].

With the implementation of self-management programmes, cost-efficiency studies have found a reduction in medical visits and healthcare costs after 12 months [31]. As in the EULAR guidelines, we recommend that patients who require lifestyle modification should receive an individualised programme, which consists of long and short-term goals, action plans, regular evaluation and follow-up, along with potential adjustments to the programme as needed [25].

Obesity is a significant and modifiable risk factor for patients with knee OA. Secondary analysis of the Intensive Diet and Exercise for Arthritis (IDEA) RCT found that there were significant dose responses to weight loss for pain (*p* = 0.01), function (*p* = 0.0006), six-minute walk distance (*p* < 0.0001), as well as physical (*p* = 0.0004) and mental (*p* = 0.03) health-related QOL (HRQOL). Long-term weight loss of 10–19.9% from baseline body weight has shown significant clinical and mechanistic benefits, and for individuals who had lost at least 10% of their baseline body weight, an additional 10% weight loss led to enhanced physical HRQOL, less pain and better function [32]. Importantly, weight loss reduces knee loading and further structural progression of knee OA [33].

Both traditional and non-traditional forms of exercise improve joint-related patient outcomes, mobility, QOL, psychological health, musculoskeletal properties, body composition, sleep and fatigue [34]. Long-term low-to-moderate or strenuous physical activity has not been associated with 10-year incident radiographic knee OA [35]. A systematic review of patients with knee OA found high- and moderate-quality evidence from 44 trials that land-based exercises such as strength training and aerobic exercise reduced pain (standardised mean difference [SMD], − 0.49; 95% CI, − 0.39 to − 0.59) and improved physical function (SMD, − 0.52; 95% CI, − 0.39 to − 0.64). Additionally, another 13 studies found high-quality evidence that land-based therapeutic exercises improved patients’ QOL (SMD, 0.28; 95% CI, 0.15 to 0.40) [36]. Traditional exercises comprising strength, flexibility, and aerobic training are beneficial in improving subjective physical function (e.g., WOMAC, Knee Injury and Osteoarthritis Outcome [KOOS]) and objective function (e.g., six-minute walk test, 30-s chair stand test) for patients with knee OA [34].

The effects of non-traditional modes of exercise such as mind-body exercises (i.e., tai chi and yoga) are deemed effective and safe for all patients with knee OA, regardless of comorbidity and have recently been recommended as part of the core treatment set [28]. A meta-analysis of nine RCTs suggested that tai chi has favourable effects on pain (SMD, − 0.79; 95% CI, − 1.19 to − 0.39; *p* = 0.0001; I^2^ = 55%), physical function (SMD, − 0.86; 95% CI, − 1.20 to − 0.52; *p* < 0.00001; I^2^ = 38%), and joint stiffness (SMD, − 0.53; 95% CI, − 0.99 to − 0.08; *p* = 0.02; I^2^ = 67%). Despite the encouraging results, the evidence that tai chi is effective in patients with OA remains limited owing to the small number of low-bias risk RCTs [37]. A recent meta-analysis of 13 RCTs reported that regular yoga training resulted in improved pain (SMD, − 0.98; 95% CI, − 1.18 to − 0.78; *p* < 0.05), reduced functional disability (SMD, − 1.83; 95% CI, − 2.09 to − 1.57; *p* < 0.05), and enhanced QOL (SMD, 0.80; 95% CI, 0.59 to 1.01; *p* < 0.05 for Short Form 36 Health Survey [SF-36] general health) in patients with knee arthritis (which included those with knee OA and rheumatoid arthritis) [38].

Soft braces/knee sleeves are commonly utilised because they are easy to use, low in cost and are recommended as a non-surgical intervention for knee OA. A systematic review with meta-analyses on the effects of soft braces/knee sleeves on self-reported pain and physical function in patients with knee OA found that there was moderate improvement in pain (SMD, 0.52; 95% CI, 0.14 to 0.89; *p* = 0.007), in favour of patients wearing a brace compared with those who did not. This effect can be present for up to 24 weeks [39].

Specific to the older population, the positive effects of a home-based exercise programme on the balance and postural stability in Malaysian seniors with OA and falls have been documented [40]. A systematic review on exercise and weight loss in older patients confirmed that international recommendations on exercise for knee OA also apply to patients aged 70–80 years [41]. All patients, including older individuals, should thus perform regular land-based exercises, if not in pain, to improve mobility and muscle strength (two to three times a week of 20–60-min sessions, depending on the type of exercise) and overweight patients who are unaffected by sarcopenia should be encouraged to lose weight. Moreover, another systematic review found that self-management education programmes in older Malaysian patients with knee OA improved health perception, treatment adherence and facilitated shared decision-making between the patient and physician [33].

#### Recommendation 4

The group recommends the use of crystalline glucosamine sulphate as background therapy for the management of knee osteoarthritis, with as needed topical non-steroidal anti-inflammatory drugs and/or oral paracetamol. Background pharmacological therapy can be initiated concurrently with prescribed physical therapies when indicated.

Symptomatic slow-acting drugs for osteoarthritis (SYSADOAs) are agents that improve symptoms and/or function and, ideally, also inhibit structural disease progression [42]. Some of the pharmacological agents regarded as SYSADOAs include glucosamine, chondroitin sulphate (CS), diacerein, and avocado/soybean unsaponifiables (ASU). The current Malaysian CPG does not prioritise the use of SYSADOAs in the treatment of knee OA [7]. A survey among primary care physicians found that only 60% of the physicians surveyed have prescribed glucosamine in their practice [43].

Three formulations of glucosamine commonly used to treat knee OA are crystalline glucosamine sulphate (CGS; a complex molecule obtained using a proprietary semi-synthetic route and stabilisation process [44]), non-crystalline glucosamine sulphate (GS) and glucosamine hydrochloride (GHCl). It is important to distinguish between the different glucosamine formulations as the clinical efficacy data is different for each preparation. Recent studies on knee OA found that only glucosamine formulations containing CGS reliably demonstrated clinical efficacy in pain and function [42, 45–49]. Given as a highly bioavailable, once daily dose of 1500 mg, CGS consistently delivered plasma levels of around 10 μmol/L – the level required to inhibit IL-1-induced expression of genes involved in the pathophysiology of joint inflammation and tissue destruction [42, 48].

**Fig. 3:**
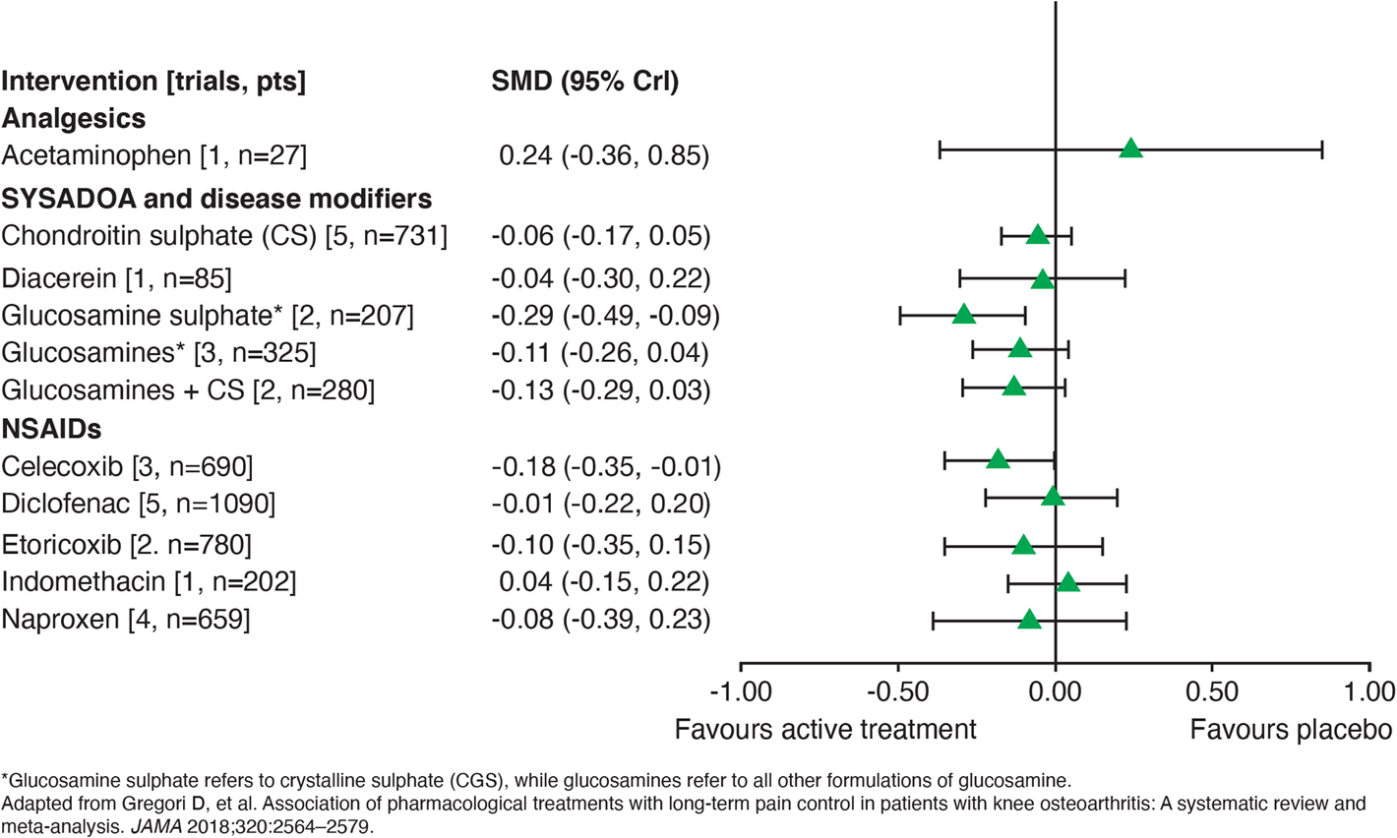
Long-term treatment effects of different pharmacological agents for knee OA that are available in Malaysia based on a meta-analysis that excluded trials at high risk of bias [50]

Pooled results from studies on glucosamine formulations found that preparations containing CGS improved pain (SMD, − 0.47; 95% CI, − 0.72 to − 0.23) and physical function (SMD, − 0.47; 95% CI, − 0.82 to − 0.12) in OA [46]; this benefit was not observed in non-CGS formulations [48]. In a network meta-analysis of long-term (> 1 year) trials of any pharmacological intervention for knee OA, only CGS was consistently effective in reducing knee OA pain (effect size [ES], 0.29; 95% CI, 0.09 to 0.49), improving physical function and joint structure changes (Fig. 3) [50]; an ES score > 0.20 is considered clinically relevant in chronic pain conditions [51].

Additionally, patients with knee OA who received CGS therapy regularly for over 3 years did not experience any significant JSN, which suggests that CGS may slow disease progression [52, 53]. Furthermore, long-term therapy with CGS reduced the need for concomitant rescue analgesia, including non-steroidal anti-inflammatory drugs (NSAIDs; odds ratio [OR], 0.64; 95% CI, 0.45 to 0.92) [54], and subsequent total knee replacement (TKR) surgery (relative risk [RR], 0.43; 95% CI, 0.20 to 0.92) compared with placebo [55].

Other glucosamine preparations such as GHCl and non-CGS preparations have been repeatedly shown to be ineffective in OA [56, 57]. The lack of appropriate stabilisation of GS impacts the availability of the active compound; an investigation of 14 commercially available preparations of GS found that the amount of the active ingredient varied from 59 to 138% of the labelled dose [42]. Glucosamine formulations in combination with chondroitin may be of variable pharmaceutical quality, but a few trials have demonstrated efficacy in knee OA. In a double-blinded RCT, patients who received GS/CS combination had significantly reduced JSN at 2 years (mean difference, 0.10; 95% CI, 0.002 to 0.20) compared with placebo [58]. In addition, a comparative analysis found that glucosamine/chondroitin could improve pain and physical function in knee and hip OA, albeit to a much lesser degree compared with oral NSAIDs [59].

Diacerein is an anthraquinone derivative with anti-inflammatory activity. A systematic review found that diacerein provided minimal pain reduction in both knee and hip OA (RR, 0.85; 95% CI, 0.72 to 0.99); while some benefit in JSN was observed, it was only clinically relevant in hip OA [60]. Although diacerein was found to be as efficacious as CGS when analysed for symptom relief in a network meta-analysis, it was also associated with more gastrointestinal (GI) adverse events (AEs) (SMD, 2.00; 95% CI, 0.69 to 5.74) compared with placebo [61]. Based on available evidence, diacerein may be used as an alternative treatment in patients with knee OA who have contraindications to NSAIDs or paracetamol [62]. However, caution has to be taken with diacerein treatment owing to an increased risk of diarrhoea (RR, 3.51; 95% CI, 2.55 to 4.83; *p* < 0.0001) compared with placebo [63].

ASU, a mixture of vegetable extracts taken from avocado and soybean oils, at doses of 300 and 600 mg/day, is associated with improvements in pain, stiffness and physical function in knee OA, and subsequently a reduced need for rescue analgesia [64]. A systematic review of RCTs found that ASU significantly reduced pain as measured by visual analogue scale assessment (weighted mean difference, − 9.64 mm; 95% CI, − 17.43 to − 1.84) in patients with knee OA compared with placebo [65]. However, ASU did not significantly improve joint structure or prevent JSN compared with placebo [66].

As safety data vary between SYSADOA agents as well as individual formulations, caution should be taken when selecting the appropriate agent for knee OA. A systematic review on the safety of SYSADOAs found that CGS, CS and ASU were not associated with any increased odds of AEs compared with placebo [67], although the evidence on ASU remains inconclusive as studies on its use have included concomitant OA medications. Diacerein has been associated with significantly increased odds of total AEs (especially GI-related AEs), including dermatological disorders and withdrawals due to AEs compared with placebo [67]. The judicious choice of SYSADOA formulation is essential to maximise clinical benefit, patient adherence and satisfaction with treatment [45].

For OA pain that occurs in spite of background CGS therapy, paracetamol is recommended for ‘as required’ therapy as the evidence for its efficacy in regular long-term use is uncertain with potential safety concerns. Paracetamol is widely regarded as an inexpensive first-line option for analgesia [50], and its use remains driven by the absence of safer therapeutic alternatives, as both oral NSAIDs and opioids are associated with potentially serious AEs [68]. Paracetamol is often used to relieve mild-to-moderate pain at doses of 600/650 (number-needed-to-treat [NNT], 4.6; 95% CI, 3.9 to 5.5) and 1000 mg (NNT, 3.8; 95% CI, 3.4 to 4.4), and is as efficacious as aspirin 600/650 mg [69]. However, there is evidence showing that paracetamol lacked both short- and long-term efficacy in improving pain and physical function in knee OA [39, 57]. In a network meta-analysis of pharmacological interventions in knee OA, paracetamol was the least efficacious intervention (ES, 0.18; 95% CI, 0.04 to 0.33), providing no clinically significant improvement on stiffness and physical function [70].

Several studies have raised concerns over the safety profile of long-term paracetamol therapy. A systematic literature review of observational studies found a considerable degree of GI, CV and renal AEs with chronic paracetamol use when dosed at the upper end of standard analgesic doses (up to 4 g/day) [68, 71]. One study found a significantly increased relative rate of mortality in patients prescribed paracetamol compared with non-users (RR, 1.14; 95% CI, 1.10 to 1.18) [72]. Moreover, consumption of paracetamol at high doses or frequencies was associated with an increased risk of myocardial infarction (MI) and stroke (RR, 1.17 and 1.30; 95% CI, 1.04 to 1.32 and 1.19 to 1.41, respectively) [72], a dose-response increase in OR of > 30% with respect to reduced estimated glomerular filtration rate, and > 0.3 mg/ dL (> 25 μmol/L) increase in serum creatinine compared with non-users [73]. However, occasional-to-moderately frequent intake of paracetamol does not appear to impair kidney function [74].

Topical NSAIDs may be added to a patient’s treatment regimen if they remain symptomatic despite background pharmacological therapy with SYSADOAs, and/or if rescue analgesia with paracetamol provides insufficient symptom relief. The short-term efficacy of topical NSAIDs, up to 2 weeks, in knee OA is well established [75–78]. However, there is no trial data to support the long-term use of topical NSAIDs in OA [75]. Data from a systematic review showed that topical NSAIDs were superior to placebo in relieving pain (SMD, − 0.30; 95% CI, − 0.40 to − 0.20) and improving function (SMD, − 0.35; 95% CI, − 0.45 to − 0.24) in OA. Of all topical NSAIDs, diclofenac patches were most effective for OA pain (SMD, − 0.81; 95% CI, − 1.12 to − 0.52) [78].

Topical NSAIDs were associated with a lower risk of systemic AEs compared with oral NSAIDs owing to lower systemic absorption [79], albeit with an increased risk of mild local skin reactions compared with placebo [76]. There was no significant difference in the odds for GI AEs between topical NSAIDs and placebo (OR, 0.96; 95% CI 0.73–1.27) [80]. Thus, topical NSAIDs may be safely used as short-term add-on pain relief, particularly in patients at increased risk of systemic AEs (e.g., older persons, multiple comorbidities).

#### Recommendation 5

The group recommends intermittent or longer cycles of oral non-steroidal anti-inflammatory drugs (NSAIDs; selective or non-selective) as advanced pharmacological treatment for patients who are still symptomatic after receiving background pharmacological and/or physical therapies. The use of oral NSAIDs should be based on an individual patient’s risk profile.

Oral NSAIDs (non-selective or cyclooxygenase-2 [COX-2] selective) may be prescribed for patients with knee OA who present with moderate-to-severe pain, or those who remain symptomatic despite background therapy with CGS, with add-on topical NSAIDs and/or oral paracetamol. Oral NSAIDs have a moderate effect in pain relief and a greater efficacy (ES, 0.29; 95% CI, 0.22 to 0.35) compared with paracetamol (ES, 0.14; 95% CI, 0.05 to 0.22) [81]. A network meta-analysis found that oral NSAIDs (celecoxib 200/400 mg, diclofenac 100/150 mg, etoricoxib 60/90 mg, ibuprofen 2400 mg, naproxen 1000 mg daily) improved pain in OA compared with placebo. Among maximally-approved doses, diclofenac 150 mg/day (ES, 0.57; 95% CI, 0.45 to 0.69) and etoricoxib 60 mg/day (ES, 0.58; 95% CI, 0.43 to 0.74) were most likely to provide the greatest efficacy on pain and physical disability [82].

Studies have shown that COX-2 selective and non-selective NSAIDs were similarly effective in managing pain [81]; the choice of NSAID depends on each individual agent’s safety profile and patients’ comorbidities [79]. Recent meta-analyses suggested that all NSAIDs have the potential for GI, CV and renal toxicity, particularly in older patients with multiple comorbidities such as hypertension, diabetes, or congestive cardiac failure [83]. Even though COX-2 selective NSAIDs were developed to avoid GI toxicity associated with non-selective NSAIDs [84], a large meta-analysis found that all NSAIDs were associated with an increased risk for GI AEs; compared with placebo, higher risks of upper GI complications were found with COX-2 inhibitors (rate ratio, 1.81; 95% CI, 1.17 to 2.81), diclofenac (rate ratio, 1.89; 95% CI, 1.16 to 3.09), ibuprofen (rate ratio, 3.97; 95% CI, 2.22 to 7.10), and naproxen (rate ratio, 4.22; 95% CI, 2.71 to 6.56) [85]. However, the risk of GI AEs may be reduced by the co-administration of a proton pump inhibitor (PPI) [79].

A meta-analysis of six RCTs found no significant differences in the rates of upper GI AEs between COX-2 selective NSAIDs and non-selective NSAIDs with concurrent PPIs (Z score, 1.67; *p* = 0.09). In addition, there was no significant difference in GI symptoms (RR, 1.10; 95% CI, 0.88 to 1.39) or CV risk (RR, 1.67; 95% CI, 0.78 to 3.59) between both groups [86]. Separately, one meta-analysis reported that non-selective NSAIDs were associated with an increased risk of acute kidney injury (pooled risk ratio, 1.58 to 2.11) compared with placebo. An elevated risk was also found for celecoxib, although this did not reach statistical significance (pooled risk ratio, 1.25; 95% CI, 0.79 to 1.97) [87].

In terms of CV risk, one meta-analysis reported that the incidence of major vascular events (i.e., non-fatal MI or stroke, death from vascular cause) was increased by the use of COX-2 inhibitors (rate ratio, 1.37; 95% CI, 1.14 to 1.66; *p* = 0.0009) and diclofenac (rate ratio, 1.41; 95% CI, 1.12 to 1.78; *p* = 0.0036) compared with placebo. The risk of hospitalisation for heart failure [HHF] is roughly doubled by all NSAID regimens [85].

When prescribing an NSAID, due consideration should be given to each patient’s GI and CV risk profile. Patients with elevated GI risk should be prescribed either a COX-2 selective NSAID, or a non-selective NSAID with concomitant PPI therapy [88]. In patients with a high GI and CV risk, the use of NSAIDs should be discouraged; if necessary, a non-selective NSAID with a lower CV risk (e.g., naproxen), may be prescribed in combination with a PPI [81].

#### Recommendation 6

The group recommends the use of intraarticular hyaluronate (IAHA) or intraarticular corticosteroids (IACS) in patients who have contraindications to oral non-steroidal anti-inflammatory drugs (NSAIDs), or if patients are still symptomatic despite the use of oral NSAIDs. In patients with knee effusion or synovitis, the use of IACS is preferred.

Intraarticular (IA) therapy is a local treatment modality that avoids AEs commonly seen with systemic agents [89]; two IA agents widely used in the treatment of knee OA are intraarticular hyaluronate (IAHA) and intraarticular corticosteroids (IACS). The proposed mechanism behind the clinical benefit seen with IAHA acts via two pathways: (i) mechanical viscosupplementation of the joint, providing greater lubrication and shock absorption, and (ii) re-establishment of joint homeostasis via endogenous HA production [90].

A Malaysian study found that IAHA, dosed three times at one-week intervals, improved pain and function in patients with knee OA at 2 weeks, with further improvements seen at both eight- and 24-weeks post-injection [91]. A meta-analysis reported that it was efficacious in pain relief after 4 weeks of treatment initiation (ES, 0.31; 95% CI, 0.17 to 0.45), reached its peak effectiveness at 8 weeks (ES, 0.46; 95% CI, 0.28 to 0.65), and exerted a residual detectable effect at 24 weeks (ES, 0.21; 95% CI, 0.10 to 0.31). Despite its slow onset, the peak efficacy of IAHA was greater than that of other analgesics commonly used in OA (i.e., paracetamol [ES, 0.13; 95% CI, 0.04 to 0.22], non-selective NSAIDs [ES, 0.29; 95% CI, 0.22 to 0.35], and COX-2 inhibitors [ES, 0.44; 95% CI, 0.33 to 0.55]) [51].

In a separate network meta-analysis, IAHA was more effective in relieving pain (ES, 0.34; 95% Cl, 0.26–0.42) compared with IA placebo at 3 months [92]. A systematic review of RCTs with a low risk of bias concluded that IAHA significantly reduced pain and improved function compared with placebo [93], and real-world evidence showed a reduction in the need for concomitant rescue analgesia, as well as the potential to delay disease progression and the need for TKR surgery [90]. A retrospective review of 45 Malaysian patients with knee OA found that 11 patients (24.4%) no longer required further treatment for the disease after receiving IAHA [94].

However, IAHA is positioned later in the treatment algorithm compared with oral NSAIDs [95], owing to the need for repeated injections by a trained healthcare provider and the inherent higher cost of treatment. The disease-modifying effects of IAHA have been validated in several retrospective database analyses which found that IAHA delayed the need for TKR by approximately 2 years; in patients who received five or more courses of IAHA, TKR was delayed by up to 3.6 years [96–98].

In terms of safety, a network meta-analysis of 18 HA products involving 13,042 patients found a very low incidence of AEs (8.5%) – most commonly transient local reactions (e.g., pain, swelling and arthralgia) which subsided rapidly. In the study, the incidence of AEs was similar between different HA formulations and was not significantly different compared with placebo [99].

IACS is indicated for short-term pain relief in patients with knee OA with evidence of synovitis. A Cochrane review found small benefits with IACS with regard to pain reduction (SMD, − 0.40; 95% CI, − 0.58 to − 0.22) and physical function (SMD, − 0.33; 95% CI, − 0.56 to − 0.09), although the evidence was of low quality [100]. Similarly, a network meta-analysis found that IACS afforded a greater reduction in pain (ES, 0.32; 95% CI, 0.16 to 0.47) – without any significant improvement in function – compared with placebo [92].

The benefits of IACS in knee OA may be limited by disease severity and structural damage. In an open-label trial examining the effects of structural factors on IACS therapy, patients with more severe knee damage – based on either magnetic resonance imaging or x-ray scans – were found to have poorer response to therapy compared with those with milder structural damage. Higher Kellgren and Lawrence and JSN scores, as well as more extensive structural damage were associated with poorer short-term outcomes [101]. Moreover, the safety of long-term IACS therapy remains a concern [102].

In a study examining the long-term effects of IACS, patients with knee OA were randomised to receive either regular IACS or IA placebo every 3 months for a total duration of 2 years. Patients in the IACS arm experienced significantly greater cartilage volume loss (between-group difference, − 0.11 mm; 95% CI, − 0.20 to − 0.03 mm) compared with those who received IA placebo with no significant difference in pain relief observed between both groups. Although the loss of cartilage did not exacerbate existing OA symptoms, it may speed up disease progression and is associated with higher rates of arthroplasty. Thus, the use of long-term, regular IACS for patients with symptomatic knee OA should be avoided [103].

A meta-analysis of seven head-to-head RCTs comparing IAHA with IACS found that the pattern of relative efficacy varied over time. From baseline to week 4, IACS appeared to be relatively more effective for pain (ES, − 0.39; 95% CI, − 0.65 to − 0.12) compared with IAHA. At week 4, there was no significant different in pain relief between both agents (ES, − 0.01; 95% CI, − 0.23 to 0.21). From week 8 and beyond, IAHA was more efficacious (efficacy at week 8: ES, 0.22; 95% CI, − 0.05 to 0.49, week 12: ES, 0.35; 95% CI, 0.03 to 0.66, and week 26: ES, 0.39; 95% CI, 0.18 to 0.59) [104]. A different meta-analysis also showed that although IACS was more effective in the short-term (i.e., two to four weeks), IAHA exhibited greater efficacy with long-term therapy (i.e., 6 months) [102].

Viscosupplementation with IAHA is only indicated in the absence of acute inflammation (i.e., no clinically detectable effusion) [105]. In patients with active knee inflammation/effusion, IACS is the preferred injectable agent [18]. Importantly, the combination of IACS and IAHA in a single injection should be avoided as clinical benefit of the combination does not last over 1 week, while there is an increased risk associated with corticosteroid use [105].

#### Recommendation 7

The group recommends the judicious use of short-term weak opioids or paracetamol/tramadol combination as rescue medication in patients who have not achieved satisfactory pain control despite the use of oral non-steroidal anti-inflammatory drugs and/or intraarticular injections.

Tramadol, a weak opioid, is recommended by the 2013 Malaysian OA CPG as an immediate step-up treatment in patients who experience inadequate pain relief with paracetamol and/or topical NSAIDs [7]. However, tramadol is seldom used in clinical practice as it is associated with an increased risk of falls, particularly among the older population [7].

In a systematic review on the safety of opioid analgesics in older adults (> 60 years) suffering from musculoskeletal pain, opioids had a small beneficial effect on pain (SMD, − 0.27; 95% CI, − 0.33 to − 0.20) and function (SMD, − 0.27; 95% CI, − 0.36 to − 0.18), which was not affected by dose or treatment duration. Of the studies included, tramadol in both immediate-release (IR) and sustained-release (SR) formulations significantly improved pain (*p* = 0.045 and *p* < 0.01, respectively) and function (*p* = 0.033 and *p* < 0.01, respectively) compared with placebo; additionally, the combination of tramadol/paracetamol significantly improved pain (*p* = 0.0095), and function (*p* = 0.0449) compared with placebo [106].

A comparative review of analgesics in knee OA found no significant differences in WOMAC pain reduction between less potent opioids (e.g., tramadol), potent opioids (e.g., hydromorphone, oxycodone), and oral NSAIDs [107]. A separate meta-analysis also showed that opioids and oral NSAIDs (both non-selective and COX-2 selective) had comparable efficacy in relieving OA pain [108]. Even though tramadol has a preferable safety profile (i.e., fewer GI and CV AEs) compared with oral NSAIDs [109], several studies have cited potential safety issues associated with the use of opioids in OA. A Cochrane review found that tramadol was associated with a greater risk of withdrawals due to AEs (RR, 2.64; 95% CI, 2.17 to 3.20) compared with placebo [109]. A recent safety meta-analysis found that the use of oral opioids – in both IR and SR formulations – was associated with an increased risk for GI, central nervous system and dermatological AEs compared with placebo [110].

The tolerability of tramadol SR may be improved via a slow upward dose titration from 50 to 100 mg, with no impact on its efficacy [111, 112]. An observational study found significantly fewer AEs (e.g., nausea, dizziness) and lower rates of treatment discontinuation due to AEs in the dose-escalation group (50 mg BD) versus the control group (100 mg BD) (5.6% vs 12.6%; *p* = 0.001), with similar efficacy [111]. An RCT involving 250 Korean patients with knee OA managed with a stable dose of NSAIDs found that the titration of tramadol/paracetamol doses was associated with improved tolerability and significantly lower discontinuation rates due to AEs compared with non-titration (10.5% vs 26.2%; *p* < 0.001); of note, both regimens displayed a similar efficacy in pain relief as measured by the Korean-WOMAC score (− 12.86 vs − 12.52, respectively) [112]. Thus, a slow upward titration of tramadol improves tolerability without compromising pain relief.

Owing to safety and potential dependency issues associated with opioids in OA, it is recommended that opioids be reserved only for short-term pain relief, after exhausting other analgesic options.

#### Recommendation 8

The group recommends the concurrent use of individual advanced pharmacological therapies (oral non-steroidal anti-inflammatory drugs, intraarticular injections, and rescue medication) when patients do not respond sufficiently to monotherapy. Patients should be assessed regularly, and treatment adjusted based on their response.

The use of concurrent therapies in knee OA is not uncommon; a large Korean study involving 2,016,516 patients with knee OA (mean age, 63.2 years) found that patients were commonly treated with combination regimens. At least 37.2% of the patients were managed on two medications (e.g., paracetamol, SYSADOA, corticosteroids), while another 16.6% of patients were managed on three medications [113]. In Malaysia, the combination of NSAIDs with other analgesics is an established practice of primary care physicians in managing knee OA [43].

A multicentre double-blinded RCT found that the addition of tramadol/paracetamol (37.5 mg/325 mg) to concomitant NSAID therapy significantly improved average daily pain intensity and pain relief scores (*p* < 0.001) compared with placebo in the treatment of OA flare pain. In the study, the addition of tramadol/paracetamol significantly improved pain (WOMAC score, − 2.56 vs − 2.02; *p* = 0.004), physical function (WOMAC score, − 2.22 vs − 1.77; *p* = 0.013), and overall well-being (WOMAC score, − 2.37 vs − 1.89; *p* = 0.008) scores compared with NSAID therapy alone. Tramadol/paracetamol was well tolerated by patients – with nausea, vomiting, and dizziness being the most common reported AEs and no serious AEs reported [114].

The chronic use of anti-inflammatory drugs (i.e., oral NSAIDs) is associated with an increased risk of upper GI AEs; which is dose dependent and increases when multiple oral NSAIDs are prescribed simultaneously. Hence, only one anti-inflammatory drug should be prescribed – at the lowest effective dose – in order to minimise the risk of serious upper GI AEs [115]. As the natural course of OA may be one of intermittent flares followed by resolution, usage of additional therapeutic options such as IA injections or other short term rescue medication in addition to oral NSAIDs for an acute flare is an option. However, the use of concurrent therapies should be for the shortest possible time and stopped when the flare has resolved.

Patients with knee OA may experience a variety of additional symptoms (e.g., mood disorders, altered sleep, impaired coping skills) resulting from pain and functional limitations associated with OA and/or other comorbidities. Thus, patients with knee OA may be treated with a combination of pharmacological and non-pharmacological interventions. A multimodal treatment plan may better address patients’ comorbidities, as well as improve overall well-being and rate of treatment success compared with management strategies using a single pharmacological agent [19].

The choice of any single or multimodal intervention in knee OA may vary over the course of the disease, or with patient and physician preferences, and should be decided as a shared decision between the patient and doctor. Physicians should exercise caution when prescribing pharmacological agents, and management should begin with treatments with the least systemic exposure or toxicity [19].

#### Recommendation 9

The group recommends knee replacement surgery (total or partial) for patients with severe/advanced knee osteoarthritis.

A network meta-analysis on surgical interventions in the management of knee OA found that osteotomy, unilateral knee arthroplasty (UKA), TKR and arthroscopic surgery effectively improved function scores of patients with knee OA. TKR displayed better long-term efficacy compared with other surgical interventions, while UKA and osteotomy were associated with better short-term efficacy [116].

Short- to medium-term studies have shown that TKR is a safe and cost-effective method for relieving pain and restoring function in patients with severe/advanced knee OA [117]. TKR is appropriate when all previous treatment modalities have failed, if the patient is severely symptomatic, or if there is significant loss in HRQOL [116–118]. TKR effectively restores mobility, improves pain, function and HRQOL in patients with severe/advance knee OA [117].

Indications for TKR include pain refractory to non-surgical treatments, significant functional impairment/disability in activities of daily living, radiographic findings (e.g., JSN, bone sclerosis), and failure of conservative treatment to manage knee OA symptoms, such as patients who remain symptomatic despite regular analgesia [119]. In addition, TKR may be considered for patients at high risk of systemic AEs associated with oral NSAIDs, owing to its preferable safety profile [120]. Following TKR, a systematic review found that there were significant intermediate- and long-term benefits with respect to both disease-specific and generic HRQOL, in particular pain (1.72; 95% CI, 0.97 to 2.46; *p* < 0.00001) and function (1.26; 95% CI, 0.87 to 1.64; *p* < 0.00001), leading to positive patient satisfaction [118].

Patient-reported outcomes in TKR may be influenced by ethnic differences [121]. A study in an Asian cohort found high levels of patient satisfaction post-TKR (92.8%), which correlated with postoperative WOMAC function scores (*p* = 0.028), postoperative WOMAC total scores (*p* = 0.040), and expectations being met (*p* = 0.033) [122].

Although TKR is a routine and safe procedure in the management of severe/advanced knee OA (survival rates at 1 year: 99%, and 10 years: 84%), up to 20% of patients have reported dissatisfaction with the overall outcome of TKR [118]. Of note, this percentage has persisted despite improvements in surgical techniques and postoperative care. A systematic review found that the degree of patient dissatisfaction post-TKR could be influenced by several key factors, including patient expectations prior to surgery, the degree of improvement in knee function, as well as pain relief following surgery [123].

In terms of safety, surgical intervention is associated with less AEs and complications compared with the use of long-term pharmacological agents, particularly NSAIDs. A systematic review found that naproxen was associated with significantly higher mortality rates (hazard ratio [HR], 3; 95% CI, 1.9 to 4.6) compared with TKR (HR, 0.79; 95% CI, 0.66 to 0.92). Additionally, GI complications were highest with the use of diclofenac (OR, 4.77; 95% CI, 3.94 to 5.76), but lowest with TKR (HR, 0.6; 95% CI, 0.49 to 0.75) [120].

## Discussion

The 2013 Malaysian CPG on the Management of Osteoarthritis, along with several other international guidelines, recommends a linear step-up approach in managing knee OA. However, pain symptoms and functional limitations experienced by arthritic patients often respond poorly to monotherapy and should instead be managed by a concurrent multimodal strategy. In the real world, simultaneously administering a combination of therapies to manage knee OA is already part of clinical practice [40, 43], and this Malaysian consensus seeks to shine light on the value of a multimodal intervention strategy in improving patient outcomes and QOL based on published evidence and clinical experience (Fig. 1).

When managing patients with knee OA whose condition has been carefully assessed, there is an overwhelming consensus that support patient education, weight management, exercise programmes, and the reduction of knee loading as the foundation of individualised treatment plan. Nonetheless, physiotherapy and non-pharmacological approaches remain significantly underutilised in Malaysia, with only 10% of physicians indicating they would refer their patients for physiotherapy [43]. Under-recognising the benefit of non-pharmacological treatment options is a barrier to optimal patient management, and this consensus emphasises multi-specialty involvement in managing knee OA as different healthcare settings have varying access to indicated treatment modalities.

Specific to pharmacological background therapy, SYSADOAs are currently not formally recommended in the Malaysian CPG. However, this consensus wishes to highlight the use of long-term SYSADOAs (specifically CGS) has been shown to consistently improve pain and physical function in knee OA; hence, their use as background pharmacological treatment for knee OA should be actively considered. In the panel’s experience, most Malaysian physicians remain unaware of the key differences between the various commercially available formulations of SYSADOAs, underlining a potential gap in awareness that this consensus aims to address.

For patients who do not respond adequately to background treatment, they may be prescribed advanced pharmacological therapies – either as a monotherapy or concurrently (if indicated). Of particular interest, the National Survey on the Use of Medicines by Malaysian consumers found that up to 73.1% of respondents admitted to medication non-compliance [124, 125]. Physicians should thus prescribe the simplest treatment regimen whenever possible to optimise treatment adherence and minimise AEs. In contrast to other existing guidelines, this consensus differentiates the role of IAHA versus IACS therapies, which are indicated in patients at greater risk for NSAID-induced AEs, or those who remain symptomatic despite the use of oral NSAIDs. In the absence of acute inflammation, the panel recommends viscosupplementation with IAHA, while IACS is the preferred choice in patients with active inflammation and/or effusion.

Overall, the choice of any single or multimodal intervention in knee OA may vary over the course of the disease, and treatment should be decided jointly between the patient and doctor. Doctors should always exercise caution when prescribing pharmacological agents, and management should begin with treatments with the least systemic exposure or toxicity.

A key feature of this consensus is the easy-to-follow one-page algorithm that illustrates the concurrent use of the recommended multimodal intervention to achieve optimal outcomes in patients with knee OA. The algorithm informs all healthcare providers of the options to combine multiple treatment modalities and step-up or step-down therapy as per patients’ needs, which confers more flexibility in patient management compared with traditional step-up algorithms advocated by other guidelines. Additionally, this consensus includes only information that is relevant to the Malaysian population (e.g., types of pharmacological agents available) to provide healthcare providers with a direct source of reference to make informed decisions about individualised patient management strategy (Fig. 3).

The strength of this consensus lies in the synthesis of well-rounded recommendations based on evidence gathered from published literature and the extensive clinical experience of the expert panel in their respective fields. The panel comprised multi-speciality physicians who actively manage knee OA in different Malaysian healthcare settings and are aware of current real-world practices that may not be reflected in existing disease management guidelines.

On the other hand, a potential limitation of this consensus is the limited number of published clinical data that are specific to the Malaysian population. Therefore, recommendations made by the panel were mostly based on international guidelines that have been adapted to the Malaysian healthcare system. Moreover, evidence on the long-term efficacy of the multimodal treatment approach advocated by the panel remains sparse. However, the panel hopes that this study will serve as an important reference point to facilitate discussions on the potential benefits of adopting a flexible multimodal approach in managing patients with knee OA.

## Conclusions

In conclusion, this consensus presents nine treatment recommendations and an algorithm for the management of knee OA. It is aimed at supporting all doctors involved in the care of patients with knee OA in Malaysia. In addition, this consensus could also be a useful guide for healthcare providers in other countries where knee OA is an increasingly common health problem.

## Data Availability

Data sharing is not applicable to this article as no datasets were generated or analysed during the current study.
